# Comparison of Adverse Events of Different Endoscopic Ultrasound-Guided Tissue Acquisition Methods: A Single-Center Retrospective Analysis

**DOI:** 10.3390/diagnostics12092123

**Published:** 2022-09-01

**Authors:** Yen-Chih Lin, Hsu-Heng Yen, Siou-Ping Huang, Kai-Lun Shih, Yang-Yuan Chen

**Affiliations:** 1 Division of Gastroenterology, Changhua Christian Hospital, Changhua 500, Taiwan; 2 College of Medicine, National Chung Hsing University, Taichung 400, Taiwan; 3 Department of Electrical Engineering, Chung Yuan Christian University, Taoyuan 320, Taiwan; 4 General Education Center, Chienkuo Technology University, Changhua 500, Taiwan; 5 Department of Hospitality Management, MingDao University, Changhua 500, Taiwan

**Keywords:** endoscopic ultrasound, pancreatic disease, fine needle biopsy

## Abstract

The efficacy of new generation endoscopic ultrasound-guided biopsy needles has been promising in recent years. Yet, comparing these needles’ diagnostic yield and safety to conventional needles is not well-known. Our study aims to compare the adverse events of endoscopic ultrasound-guided tissue acquisition (EUS-TA) with different types of needles, including FNA needles, FNB needles with a Franseen tip and FNB needles with a reverse bevel. Furthermore, we will analyze the risk factors, including tumor vascularity, different needle types, and the underlying disease, which may impact the safety of the procedures. From May 2014 to December 2021, 192 consecutive EUS-TAs were performed on pancreatic and peripancreatic lesions in our hospital using different types of FNA and FNB needles. We retrospectively reviewed the data and identified the risk factors for EUS-TA-related complications. As a result, the hypervascular tumor is a significant risk factor for adverse events in our multivariate analysis, with an odds ratio of 4.96 (95% CI 1.33–18.47), while liver cirrhosis is one of the risk factors for adverse events during EUS-TA, with an odds ratio of 5.3 (95% CI 1.1–25.6). However, the risk of adverse events did not increase using Franseen-tip needles, compared to conventional FNA or FNB needles with a reverse bevel. In conclusion, we must be more cautious in patients with liver cirrhosis and hypervascular tumors, such as pancreatic neuroendocrine tumors, when performing EUS-guided tissue acquisition.

## 1. Introduction

Endoscopic ultrasound-guided tissue acquisition, including endoscopic ultrasound-guided fine-needle aspiration (EUS-FNA) and endoscopic ultrasound-guided fine-needle biopsy (EUS-FNB), has emerged as an accurate and safe modality in the diagnosis of pancreatic masses as well as one of the valuable diagnostic tools for intra-abdominal masses [1]. Although the specimen obtained from EUS-FNA is fragile and often insufficient for pathological evaluation, EUS-FNA has acceptable sensitivity and specificity for diagnosis with multiple needle passes [2]. In recent years, needles dedicated to fine-needle biopsy have been modified. As a result, more intact tissue sampling has become possible using this new generation of fine-needle biopsy devices. The accuracy of ultrasound-guided fine-needle biopsy without rapid on-site evaluation (ROSE) is not inferior to ultrasound-guided fine-needle aspiration with the aid of ROSE, and EUS-FNB alone is associated with fewer needle passes, shorter procedure time, and excellent histological yield [3,4].

The EUS-FNB needles’ designs are divided into side fenestrations, Franseen tip, and Fork tip. Theoretically, these designs could carry a higher risk for tissue trauma than conventional aspiration needles, leading to a higher risk of bleeding, pancreatitis, or other adverse events. Some studies compared the efficacy and safety of EUS-FNA with EUS-FNB. Still, limited data exist regarding the impact of different FNB needles and the patient’s comorbidity, which may be associated with procedure-related adverse events. Therefore, our study aims to compare the adverse events of Endoscopic ultrasound-guided tissue acquisition (EUS-TA) with different types of needles, namely FNA needles, FNB needles, Franseen-tip needles, and FNB needles with reverse bevel [5,6,7]. Furthermore, we will analyze the risk factors, including tumor vascularity, different needle types, and the underlying disease, that may impact the safety of the procedures. 

## 2. Materials and Methods

### 2.1. Patients and Data Preparation

From May 2014 to December 2021, our hospital performed consecutive EUS-TAs on pancreatic and peripancreatic masses. After excluding simple fluid analysis (N = 21) and missing data (N = 6), a total of 192 procedures of EUS-FNA/FNB on pancreatic and peripancreatic lesions was performed on 185 patients in our hospital (Table 1). We retrieved the data of every patient undergoing EUS-TA registered in the database from 2014 and retrospectively reviewed the medical file, including the complications and final diagnosis. We checked the platelet count and prothrombin time before EUS-TA, and all enrolled patients had platelet count > 50,000/μL, without prolongation of prothrombin time. Those on anticoagulant therapy were instructed to discontinue the medication before the procedure, according to ESGE guidelines [8]. In addition, before undergoing EUS-TA, all patients provided written informed consent. The local ethics committee approved the study in our hospital (IRB no. 220423). 

### 2.2. EUS-TA Procedures 

The physician who performed EUS-TA procedures in this study had performed more than 200 EUS procedures (including 120 EUS-TA procedures) in a high-volume center before the study period. All the patients were placed in the left lateral position, and conscious sedation was performed using either intravenous pethidine hydrochloride (35 mg) or intravenous fentanyl (50 µg), along with intravenous midazolam (5 mg).

All EUS-TA procedures were completed using a curved linear echoendoscope (GF-UCT260; Olympus Medical Systems, Tokyo, Japan). The endoscopic ultrasound systems we used were EU-ME1 (2014–2020) and EU-ME2 (since 2020) (Olympus Medical Systems, Tokyo, Japan). 

The needle was selected according to the judgment of the operator. For FNA, 19 G, 22 G, or 25 G needle Expect (Boston Scientific, Marlborough, MA, USA); EZ-shot 2 (Olympus Medical Systems, Hamburg, Germany); or EchoTip Ultra (Cook Medical, Daniels Way Bloomington, IN, USA) were selected. For FNB, 22 G or 25 G Acquire (Boston Scientific); 22 G or 25 G Topgain (Mediglobe, Tempe, AZ, USA); and 22 G or 25 G EchoTip Procore (Cook Medical) were used. 

After the needle was inserted into the target lesion, the stylet was removed. For FNA needles, to and fro motions were performed 20 times using negative pressure in the syringe. For the FNB needle, to and fro motions were performed 10 times, with the so-called “slow pull method” or without negative pressure, depending on the needle size and the vascularity of the target. Since rapid on-site evaluation (ROSE) is not available in our institution, we applied the method, called “macroscopic on-site quality evaluation” (MOSE), right after the method was first reported in 2015 by a Japanese medical team [9]. As a result, the final diagnosis of pancreatic masses was confirmed by a combination of smear cytology, histopathology, surgery, imaging, and clinical follow-up for more than six months.

### 2.3. Assessment of Adverse Events and Variables 

After the procedure, the medical team confirmed the symptoms and physical findings on the day following the procedure and followed up on the case at the outpatient department one week after discharge. We arranged diagnostic imaging such as abdominal sonography or computed tomography (CT), if the patients had any complaints suggestive of acute pancreatitis or peritonitis. According to the Atlanta criteria, pancreatitis was diagnosed based on abdominal pain, abdominal sonography, or CT findings and elevated pancreatic enzyme greater than three times normal limits. Besides, gastrointestinal bleeding was defined as a drop in the hemoglobin level by 2 g/dL, as compared with the baseline levels and clinical signs of bleeding, along with any events undergoing endoscopic hemostasis after tissue acquisition. If a large coagulum on the puncture site was noted right after EUS-TA, we also defined it as minor (mild) bleeding. The severity of the adverse events was defined according to a workshop held by the American Society for Gastrointestinal Endoscopy (ASGE), published in 2010 [10]. 

### 2.4. Statistics 

Data are expressed as n (%) and median (interquartile range, IQR) (range). The one-sample Kolmogorov–Smirnov test examined the distribution of continuous variables. In comparing the three groups (conventional FNA needles, FNB with Franseen-tip needles, and FNB needles with a reverse bevel), categorical variables were compared using the chi-squared test or Fisher’s exact test with Bonferroni correction, and continuous variables were compared using Kruskal–Wallis test. In the comparisons between the two groups (with/without complication), categorical variables were compared using the chi-squared test or Fisher’s exact test, and continuous variables were compared using the Mann–Whitney U test (continuous data), as appropriate. To enter the multivariate adjustment model 1 and model 2, a multivariate logistic regression analysis was used to analyze factors associated with the complication and selected variables with a *p*-value < 0.2 from the crude model. Moreover, model 2 used the stepwise backward elimination procedure. The results were statistically significant if the *p*-value was <0.05 for all tests. All statistical data were analyzed using IBM SPSS version 22.0 (IBM Corp., Armonk, NY, USA).

## 3. Results

During the study period, 192 procedures of EUS-TA were performed. The overall complication rate was 6.7% (13), and the overall gastrointestinal bleeding rate was 4.2% (8). Among them, six cases with a gastrointestinal bleeding event were mild or moderate bleeding, while two cases needed blood transfusions or prolonged admission. Besides those hemorrhage episodes, there were one case of mild pancreatitis and two cases of fever after tissue acquisition. In two patients, duodenal perforation occurred after EUS-TA, so the patients underwent emergent surgery (case 8 and case 11).

The baseline characteristics are listed in Table 1. Since perforation is related to scope manipulation rather than EUS-FNA/FNB-related complications, the two cases of perforation were excluded from our statistical analysis. We divided the needle type into three groups, which were the FNA needle (N = 76), FNB needle with a Franseen tip (N = 95), and FNB needle with a reverse bevel (N = 19). In these three groups, there was no significant difference between patient factors (age, gender, comorbidity), tumor factors (tumor size, location, vascularity, cystic component), or technique factors such as needle passes and sampling route. However, we tended to select a 22 G needle when we performed FNB with a Franseen-tip needle (69.5% vs. 43.4%, *p* = 0.002) and to select a 25 G needle when we performed FNA with a conventional FNA needle (55.3% vs. 30.5%, *p* = 0.003). In our institution, a 19 G FNA needle was selected when we performed EUS-guided pancreatic pseudocyst drainage or other EUS-guided local treatment such as fluid analysis or ethanol injection [11]. In terms of adverse events, there was no adverse event in the group using an FNB needle with a reverse bevel. At the same time, there were six cases (7.9%) of adverse events in the FNA group and five cases (5.3%) of adverse events in the group using an FNB needle with a Franseen tip (*p* = 0.482).

The variables were examined in 190 patients who underwent EUS-TA for the pancreatic and peripancreatic masses to identify the risk factors of adverse events during the procedures. They are patient factors such as age, gender, comorbidity (compensated liver cirrhosis and end-stage renal disease), tumor factors such as tumor size, tumor vascularity (hypervascular or hypovascular), cystic component in the mass, and technique factors such as needle types (FNA needles, FNB with a Franseen tip, or FNB with a reverse bevel), route of sampling, size of the needle, and the number of needle passes. The diagnosis of liver cirrhosis in our hospital was made by gastroenterologists based on clinical examination, imaging findings (CT, MRI, abdominal sonography), and laboratory findings suggestive of liver cirrhosis and portal hypertension. Besides, the vascularity of the tumors was evaluated by contrast CT/MRI and Doppler EUS. A hypervascular lesion was defined as a hyperdense/hyperechoic mass compared to adjacent pancreas parenchyma on either contrast CT, contrast MRI, or doppler EUS. End-stage renal disease was defined as having a glomerular filtration rate of less than 15 mL/min. Univariate analysis showed that the incidence of procedural complications was significantly high in patients with old age (*p* = 0.039), liver cirrhosis (*p* = 0.024), and hyper-vascular tumors (*p* = 0.010) (Table 2). Multivariate analysis identified hypervascular tumors (OR 4.96, 95% CI 1.33–18.47) and liver cirrhosis (OR 5.3, 95% CI 1.1–25.6) as independent risk factors of adverse events (Table 3). All the details of adverse events are listed in Table 4.

## 4. Discussion

Our study’s overall complication rate associated with EUS-TA was 5.8%, slightly higher than that reported in previous studies [7,12,13,14,15,16]. For example, Hamada et al. [13] reported a severe bleeding rate of 0.23% in 212 patients undergoing EUS-FNA. In addition, Akio Katanuma et al. reported a complication rate of 3.4% in a retrospective study in 2013 [17]. Unlike prior studies, our study enrolled patients with compensated liver cirrhosis and end-stage renal disease, which may explain the slightly higher complication rate. Among our adverse events, the majority were bleeding events, with an overall bleeding rate of 4.2% (N = 8). However, most of them were mild to moderate in severity (6/8), and severe bleeding events requiring prolonged admission or ICU care were observed in two cases (2/8).

Peter Vilmann first performed EUS-TA for the cytologic diagnosis of a pancreatic lesion in 1991 [18].This technique has now become the standard method for tissue acquisition from pancreatic and peripancreatic lesions [14,19]. A recent network meta-analysis demonstrated better accuracy with 22-gauge FNB needles than conventional 22-gauge FNA needles [20], but it did not compare the performance between different types of FNB needles, namely, side-fenestrated needles, Franseen-tip needles, and Fork-tip needles. About this issue, another network meta-analysis in 2022 demonstrated the higher accuracy of Franseen-tip needles and Fork-tip needles than reverse-bevel needles and conventional FNA needles [21,22], especially in the absence of ROSE [3].

However, the safety of novel FNB needles, compared to conventional FNA needles and reverse-bevel needles, has rarely been discussed. A retrospective study in 2021 revealed that the use of FNB needles (most of them Franseen-tip needles and Fork-tip needles) and lower platelet count were two parameters associated with minor bleeding after EUS-TA [23]. In our multivariate analysis, there is no statistically significant difference between Franseen-tip needles, conventional FNA needles, and reverse-bevel needles regarding adverse events. The difference between our study and that prior study may be due to the high proportion of antithrombotic agents used in that study (29.8%, in the solid tumor group). Similarly, a recent meta-analysis comparing the efficacy and safety of EUS-FNA and EUS-FNB also revealed a rare occurrence of AE, with no difference between FNA and FNB. However, due to the rarity of AE and variations in reporting, that meta-analysis was unable to provide meaningful analysis regarding specific complications [24,25]. Given the conflicting result compared to prior studies, we considered that a prospective study enrolling a more significant number of patients is still needed to determine the risk factors of adverse events during EUS-TA.

**Figure 1:**
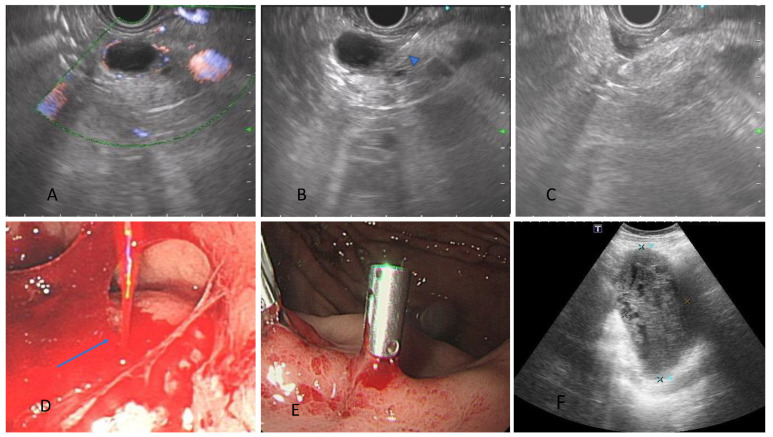
A case of severe bleeding after EUS-FNB in a patient with cystic pancreatic neuroendocrine tumor. (**A**) A cystic lesion 1.8 cm in pancreatic tail, with blood flow noted in the rim. (**B**) We punctured the thickened wall of the cystic lesion using 22 G Franseen-tip needle without penetrating intervening vessels (arrowhead). (**C**) Some fluid accumulation was visualized around the pancreas after tissue acquisition. (**D**) After shifting to the endoscopic view, a spurting vessel was noted (arrow). (**E**) Two hemoclips were applied right after EUS-FNB, and hemostasis was achieved. (**F**) Follow-up abdominal sonography 1 month later showed a huge intra-abdominal hematoma, 14 cm × 5 cm.

Hypervascular tumors are important risk factors for adverse events in our multivariate analysis, with an odds ratio of 4.96 (95% CI 1.33–18.47). When we perform EUS-TA, pancreatic neuroendocrine and gastrointestinal stromal tumors are the most common hypervascular tumors we may encounter. Small pNETs are almost hypervascular tumors, and the risk of bleeding from these tumors is increased compared with hypovascular tumors. When bleeding occurs around the pancreatic parenchyma after EUS-FNA/FNB, it may present as both upper gastrointestinal or intra-abdominal bleeding (case 13 in Table 4; Figure 1). A retrospective study by Akio Katanuma et al. demonstrated that pNET is the most critical risk factor for an adverse event, with an odds ratio of 36.5, compared to benign lesions using FNA needles (14). Our study showed that hypervascular tumors remained the most crucial risk factor for an adverse event in the era of EUS-FNB. Recently, EUS-guided through-the-needle biopsy (EUS-TTNB) has been developed and proven to be a safe and effective tool in EUS-guided tissue acquisition, especially when we encountered cystic lesions in a pancreas with suspicion of a cystic neoplasm or a malignant change in a pancreatic cyst. In the cystic neoplasm, such as our case 13, we should keep in mind that EUS-guided through-the-needle biopsy is one of the choices because this method can avoid a hemorrhage into the retroperitoneal cavity after tissue acquisition [26]. However, a recent study has identified the risk factors and risk classes of an adverse event after EUS-TTNB, and it becomes crucial to select patients before EUS-TTNB to optimize the benefit and risk of this procedure [27]. Besides, a recent meta-analysis demonstrated that routine use of hemostatic powder is helpful for patients with upper gastrointestinal bleeding, suggestive of the possibility of applying this method to EUS-TA for a subepithelial lesion [28].

Our study demonstrated that liver cirrhosis is one of the risk factors for adverse events during EUS-TA, with an odds ratio of 5.3 (95% CI 1.1–25.6). Reports about the adverse events of cirrhotic patients in the English literature are rare. A retrospective study of 692 patients who underwent ERCP revealed that overall complications, including pancreatitis and bleeding, were higher in those with cirrhosis than those without cirrhosis (*p* = 0.015) [29], suggesting that liver cirrhosis is a significant risk factor during a pancreaticobiliary endoscopic procedure. On the other hand, a study of EUS-guided liver biopsy that included 15.8% cirrhotic patients showed that adverse events were uncommon (1.8%), most of which were self-limited [30]. However, as shown in our data, it is reasonable to say that liver cirrhosis is a risk factor for an adverse event during EUS-TA, since a low platelet count is common in cirrhotic patients, and thrombocytopenia itself is a well-known risk factor of bleeding during EUS-TA [23].

The study has some limitations. First of all, due to the retrospective design and relatively small case number, selection bias may occur [31,32,33,34]. However, although our study was retrospective, all the patients undergoing EUS-TA were registered in a database every time we performed these procedures. Therefore, we can ensure the accuracy of our database. Second, this study only enrolled pancreatic and peripancreatic tumors, while pseudocysts and subepithelial tumors (SET) in the gastrointestinal tract were excluded. Hence, the result of our study cannot be valid for SET. Third, although the use of antithrombotic agents before EUS-TA was based on the ESGE guideline, we had no detailed information about all the drug history before the patients were admitted. Finally, since the period of data analysis is long (2014–2021), there was a learning curve for both the examiner and the cytopathologist, which may influence our data analysis [35,36].

## 5. Conclusions

In conclusion, different needle types (conventional FNA, Franseen-tip, or reverse-bevel needles) did not impact the safety of EUS-TA in our study. On the contrary, hypervascular tumors and cirrhotic patients were risk factors for adverse events during EUS-TA, so we must be more cautious in patients with such risk factors.

## Figures and Tables

**Table 1:** Baseline characteristics of the patients categorized by different needle types.

	Total (N = 190)	FNA (N = 76)	FNB (2) * (N = 95)	FNB (3) ** (N = 19)	*p*-Value			
					Comparison			
					Between Groups	Post Hoc Tests		
						FNA vs. FNB (2)	FNA vs. FNB (3)	FNB (2) vs. FNB (3)
Age, years, median (IQR) (range)	64 (55–72) [27–88]	66.5 (57.5–73) [32–88]	63 (53–71) [27–82]	63 (50–68) [38–81]	0.205	--	--	--
Gender, male, n (%)	108 (56.8%)	48 (63.2%)	50 (52.6%)	10 (52.6%)	0.357	--	--	--
Liver cirrhosis, n (%)	12 (6.3%)	5 (6.6%)	6 (6.3%)	1 (5.3%)	1.000	--	--	--
ESRD, n (%)	6 (3.2%)	3 (3.9%)	3 (3.2%)	0 (.0%)	1.000	--	--	--
Tumor location, n (%)					0.417	--	--	--
Head	93 (48.9%)	38 (50.0%)	49 (51.6%)	6 (31.6%)	--	--	--	--
Body or tail	80 (42.1%)	33 (43.4%)	36 (37.9%)	11 (57.9%)	--	--	--	--
Peripancreas	17 (8.9%)	5 (6.6%)	10 (10.5%)	2 (10.5%)	--	--	--	--
Tumor size, cm, median (IQR) (range)	3 (2.1–3.7) [0–12]	2.9 (2.2–4) [0.94–7.6]	3 (2–3.6) [0–12]	3.2 (2.5–4.2) [1–6.3]	0.414	--	--	--
Tumor vascularity, n (%)					0.596	--	--	--
Hypovascular lesion	163 (85.8%)	64 (84.2%)	81 (85.3%)	18 (94.7%)	--	--	--	--
Hypervascular lesion	27 (14.2%)	12 (15.8%)	14 (14.7%)	1 (5.3%)	--	--	--	--
Cystic component in the mass, n (%)	21 (11.1%)	9 (11.8%)	11 (11.6%)	1 (5.3%)	0.888	--	--	--
Needle size, n (%)					0.001	0.002	1.000	0.132
Other (>22 G)	2 (1.1%)	1 (1.3%)	0 (.0%)	1 (5.3%)	0.090	1.000	1.000	0.500
22 G	108 (56.8%)	33 (43.4%)	66 (69.5%)	9 (47.4%)	0.002	0.002	1.000	0.191
25 G	80 (42.1%)	42 (55.3%)	29 (30.5%)	9 (47.4%)	0.004	0.003	1.000	0.465
Needle pass, times, median (IQR) (range)	2 (2–3) [1–5]	2 (2–3) [1–5]	2 (2–3) [1–4]	3 (2–3) [1–3]	0.646	--	--	--
Needle pass, times, n (%)					0.261	--	--	--
<2 or =2	110 (57.9%)	43 (56.6%)	59 (62.1%)	8 (42.1%)	--	--	--	--
>3 or =3	80 (42.1%)	33 (43.4%)	36 (37.9%)	11 (57.9%)	--	--	--	--
Route of sampling, n (%)					0.420	--	--	--
Trangastric	107 (56.3%)	44 (57.9%)	50 (52.6%)	13 (68.4%)	--	--	--	--
Transduodenal	83 (43.7%)	32 (42.1%)	45 (47.4%)	6 (31.6%)	--	--	--	--
Any adverse event (11), n (%)	11 (5.8%)	6 (7.9%)	5 (5.3%)	0 (.0%)	0.482	--	--	--
Bleeding events (8), n (%)	8 (4.2%)	5 (6.6%)	3 (3.2%)	0 (.0%)	0.478	--	--	--

* FNB (2): Franseen-tip needle; ** FNB (3): needle with a reverse bevel.

**Table 2:** Univariate analysis for the risk factors of EUS-TA-related complications.

	Total (N = 190)	With Complication (N = 11)	Without Complication (N = 179)	*p*-Value
Age, years, median (IQR) [range]	64 (55–72) [27–88]	72 (67–77) [27–86]	63 (55–72) [30–88]	0.039
Gender, Male, n (%)	108 (56.8%)	7 (63.6%)	101 (56.4%)	0.76
Liver Cirrhosis, n (%)	12 (6.3%)	3 (27.3%)	9 (5.0%)	0.024
ESRD, n (%)	6 (3.2%)	1 (9.1%)	5 (2.8%)	0.304
Tumor location, n (%)				0.402
Head	93 (48.9%)	89 (49.7%)	4 (36.4%)	--
Body or tail	80 (42.1%)	75 (41.9%)	5 (45.5%)	--
Peripancreas	17 (8.9%)	15 (8.4%)	2 (18.2%)	--
Needle type, n (%)				0.482
FNA	76 (40.0%)	6 (54.5%)	70 (39.1%)	--
FNB(2) *	95 (50.0%)	5 (45.5%)	90 (50.3%)	--
FNB(3) **	19 (10.0%)	0 (0.0%)	19 (10.6%)	--
Needle size, n (%)				0.220
Other (>22G)	2 (1.1%)	0 (0.0%)	2 (1.1%)	--
22G	108 (56.8%)	9 (81.8%)	99 (55.3%)	--
25G	80 (42.1%)	2 (18.2%)	78 (43.6%)	--
Route of sampling, n (%)				0.759
Transgastric	107 (56.3%)	7 (63.6%)	100 (55.9%)	--
Transduodenal	83 (43.7%)	4 (36.4%)	79 (44.1%)	--
Tumor Size, cm, median (IQR) [range]	3 (2.1–3.7) [0–12]	2.6 (1.1–5) [1–7]	3 (2.1–3.7) [0–12]	0.559
Tumor vascularity, n (%)				0.010
Hypovascular lesion	163 (85.8%)	6 (54.5%)	157 (87.7%)	--
Hypervascular lesion	27 (14.2%)	5 (45.5%)	22 (12.3%)	--
Cystic component (present or absent), n (%)	21 (11.1%)	19 (10.6%)	2 (18.2%)	0.349
Needle pass, times, median (IQR) [range]	2 (2–3) [1–5]	2 (2–2) [1–3]	2 (2–3) [1–5]	0.087
Needle pass, times, n (%)				0.123
<2 or =2	110 (57.9%)	9 (81.8%)	101 (56.4%)	--
>3 or =3	80 (42.1%)	2 (18.2%)	78 (43.6%)	--

* FNB (2): Franseen-tip needle ** FNB (3): needle with a reverse bevel.

**Table 3:** Multivariate analysis for the risk factors of EUS-TA-related complication.

	Crude		Adjusted Model 1		Adjusted Model 2	
Variables	OR (95%CI)	*p*-Value	OR (95%CI)	*p*-Value	OR (95%CI)	*p*-Value
Age, years	1.05 (0.99–1.11)	0.11	1.04 (0.98–1.1)	0.218		
Gender (Male)	1.35 (0.38–4.78)	0.640				
Liver Cirrhosis	7.08 (1.6–31.32)	0.010	7.22 (1.24–42.01)	0.028	5.3 (1.1–25.6)	0.038
ESRD	3.48 (0.37–32.68)	0.275				
Tumor location, n (%)						
Head	1 [Reference]					
Body or tail	1.48 (0.38–5.72)	0.567				
Peripancreas	2.97 (0.5–17.65)	0.232				
Needle type						
FNB(2) *	1 [Reference]					
FNA	1.54(0.45–5.26)	0.489				
FNB(3) **	Unestimated	0.998				
Needle size						
G22	1 [Reference]		1 [Reference]			
G25	0.28 (0.06–1.34)	0.112	0.23 (0.04–1.31)	0.098		
Other (22G)	Unestimated	0.999	Unestimated	0.999		
Route of sampling						
Transgastric	1 [Reference]					
Transduodenal	0.72 (0.2–2.56)	0.615				
Tumor Size, cm	0.98 (0.64–1.5)	0.933				
Tumor vascularity						
Hypovascular lesion	1 [Reference]		1 [Reference]		1 [Reference]	
Hypervascular lesion	5.95 (1.67–21.13)	0.006	3.75 (0.94–14.98)	0.062	4.96 (1.33–18.47)	0.017
Cystic component (present or absent), n (%)	1.87 (0.38–9.31)	0.444				
Needle pass, times, n (%)						
<2 or =2	1 [Reference]		1 [Reference]			
>3 or =3	0.29 (0.06–1.37)	0.118	0.33 (0.06–1.73)	0.188		

* FNB (2): Franseen-tip needle ** FNB (3): needle with a reverse bevel.

**Table 4:** Patient list of an adverse event associated with EUS-TA.

	Age	Gender	Final Diagnosis	Tumor Location	Tumor Size (cm)	Route of Needle Pass	Needle Size (Gauge)	Number of Passes	Needle Type	Tumor Vascularity	LC	ESRD	Adverse Event	Severity
1	74	M	GIST	Body to tail	7	Transgastric	22	2	FNA	Hypervascular	No	No	Bleeding	Mild
2	79	F	Chronic inflammation	Peripancreas	4.40	Transgastric	22	3	FNA	Hypovascular	No	No	Fever, cause?	Moderate
3	27	M	Chronic pancreatitis	Body to tail	1	Transgastric	22	2	FNB	Hypovascular	No	No	Acute pancreatitis	Mild
4	86	F	Neuroendocrine tumor	Head	1.10	Tranduodenal	22	2	FNA	Hypervascular	No	No	Bleeding	Mild
5	61	M	Chronic pancreatitis	Head	2.6	Tranduodenal	25	2	FNA	Hypovascular	Yes	No	Bleeding	Mild
6	72	M	Neuroendocrine tumor	Head	1.2	Tranduodenal	22	1	FNA	Hypervascular	No	No	Bleeding	Mild
7	68	F	Pancreatic ductal adenocarcinoma	Head	2.9	Tranduodenal	22	2	FNA	Hypovascular	No	No	Bleeding	Mild
8	72	F	Autoimmune pancreatitis	Head	2.2	Tranduodenal	25	2	FNA	Hypovascular	No	No	Duodenal performation	Severe
9	67	M	Neuroendocrine tumor	Body to tail	1.00	Transgastric	25	2	FNB	Hypervascular	Yes	No	Bleeding	Moderate
10	77	F	Pancreatic ductal adenocarcinoma	Body to tail	5.1	Transgastric	22	2	FNB	Hypovascular	No	No	Fever, cause?	Severe
11	70	F	Pancreatic ductal adenocarcinoma	Body to tail	3s	Transgastric	22	3	FNB	Hypovascular	No	No	Duodenal performation	Severe
12	69	M	Poorly differentiated carcinoma	Peripancreas	5	Transgastric	22	2	FNB	Hypovascular	Yes	No	Bleeding	Severe
13	76	M	Neuroendocrine tumor	Body to tail	1.8	Transgastric	22	3	FNB	Hypervascular	No	Yes	Bleeding	Severe

M: male; F: female, LC: liver cirrhosis, ESRD: End stage renal disease.

## Data Availability

The datasets generated and/or analyzed during the current study are not publicly available, but these may be requested from the corresponding author upon reasonable request.
